# Prevalence and associated risk factors of *Leishmania* infection among immunocompetent hosts, a community-based study in Chiang Rai, Thailand

**DOI:** 10.1371/journal.pntd.0009545

**Published:** 2021-07-12

**Authors:** Pamornsri Sriwongpan, Supalert Nedsuwan, Jidapa Manomat, Sakarn Charoensakulchai, Kittiphat Lacharojana, Jamnong Sankwan, Natheeporn Kobpungton, Taweesak Sriwongpun, Saovanee Leelayoova, Mathirut Mungthin, Suradej Siripattanapipong, Toon Ruang-areerate, Tawee Naaglor, Theethach Eamchotchawalit, Phunlerd Piyaraj

**Affiliations:** 1 Department of Public Health, School of Health Science, Mae Fah Luang University, Chiang Rai, Thailand; 2 Social and Preventive Medicine Department, Chiangrai Prachanukroh Hospital, Ministry of Public Health, Chiang Rai, Thailand; 3 Department of Microbiology, Faculty of Science, Mahidol University, Bangkok, Thailand; 4 Department of Parasitology, Phramongkutklao College of Medicine, Bangkok, Thailand; 5 Wiang Kaen Hospital, Ministry of Public Health, Chiang Rai, Thailand; 6 Chiangrai Provincial Livestock Office, Ministry of Agriculture and Cooperatives, Chiang Rai, Thailand; 7 Anatomical Pathology Department, Chiangrai Prachanukroh Hospital, Ministry of Public Health, Chiang Rai, Thailand; 8 Vector Borne Disease Control Center 1.3, Chiang Rai, Thailand; Institut Pasteur de Tunis, TUNISIA

## Abstract

**Background:**

*Leishmaniasis* is an emerging infectious disease reported in the north and south of Thailand of which patients with HIV/AIDS are a high risk group for acquiring the infection. A lack of information regarding prevalence, and the risk association of *Leishmania* infection among asymptomatic immunocompetent hosts needs further investigation. Information on potential vectors and animal reservoirs in the affected areas is also important to control disease transmission.

**Methods:**

An outbreak investigation and a cross-sectional study were conducted following one index case of cutaneous leishmaniasis (CL) caused by *L*. *martiniquensis* in an immunocompetent male patient reported in August 2015, Chiang Rai Province, Thailand. From September to November 2015, a total of 392 participants at two study areas who were related to the index case, 130 students at a semi-boarding vocational school and 262 hill tribe villagers in the patient’s hometown, were recruited in this study. The nested internal transcribed spacer 1-PCR (ITS1-PCR) was performed to detect *Leishmania* DNA in buffy coat, and nucleotide sequencing was used to identify species. Antibody screening in plasma was performed using the Direct Agglutination Test (DAT), and associated risk factors were analyzed using a standardized questionnaire. Captured sandflies within the study areas were identified and detected for *Leishmania* DNA using nested ITS1-PCR. Moreover, the animal reservoirs in the study areas were also explored for *Leishmania* infection.

**Results:**

Of 392 participants, 28 (7.1%) were positive for *Leishmania* infection of which 1 (4.8%) was *L*. *martiniquensis*, 12 (57.1%) were *L*. *orientalis* and 8 (38.1%) were *Leishmania* spp. Of 28, 15 (53.6%) were DAT positive. None showed any symptoms of CL or visceral leishmaniasis. Risk factors were associated with being female (adjusted odds ratio, AOR 2.52, 95%CI 1.01–6.26), increasing age (AOR 1.05, 95%CI 1.02–1.08), having an animal enclosure in a housing area (AOR 3.04, 95%CI 1.13–8.22), being exposed to termite mounds (AOR 3.74, 95%CI 1.11–12.58) and having domestic animals in a housing area (AOR 7.11, 95%CI 2.08–24.37). At the semi-boarding vocational school, six *Sergentomyia gemmea* samples were PCR positive for DNA of *L*. *orientalis* and one *S*. *gemmea* was PCR positive for DNA of *L*. *donovani/L*. *infantum*. Additionally, one *Phlebotomus stantoni* was PCR positive for DNA of *L*. *martiniquensis*, and one black rat (*Rattus rattus*) was PCR positive for DNA of *L*. *martiniquensis*.

**Conclusion:**

This information could be useful for monitoring *Leishmania* infection among immunocompetent hosts in affected areas and also setting up strategies for prevention and control. A follow-up study of asymptomatic individuals with seropositive results as well as those with positive PCR results is recommended.

## Author summary

This is the first community-based study investigating the prevalence and associated risk factors of *Leishmania* infection among immunocompetent individuals, demonstrating the situation of *Leishmania* infection in endemic areas of Thailand. An outbreak investigation and a cross-sectional study were conducted following one index case of cutaneous leishmaniasis (CL) caused by *L*. *martiniquensis* in an immunocompetent male patient reported in August 2015, Chiang Rai Province, Thailand. Of 392 participants, 28 (7.1%) were positive for *Leishmania* infection of which 1 (4.8%) was *L*. *martiniquensis*, 12 (57.1%) were *L*. *orientalis* and 8 (38.1%) were *Leishmania* spp. The factors associated with *Leishmania* infection included being female, increasing age, having an animal enclosure in a housing area, being exposed to termite mounds and having domestic animals in a housing area. This information revealed the first burden and risk factors of *Leishmania* infection among immunocompetent individuals in Thailand and highlighted the urgent need for effective interventions to prevent and control the disease in high risk populations in endemic areas.

## Introduction

Leishmaniasis is a neglected tropical disease caused by a flagellate protozoa, genus *Leishmania* [1]. The causative agents for leishmaniasis mainly belong to the subgenus *L*. *(Leishmania)* and subgenus *L*. *(Viannia)*. In 2018, Leishmania species in the *L*. *enriettii* complex were assigned to a new subgenus *L*. *(Mundinia)*. *L*. *(Mundinia)* are responsible for human and animal leishmaniasis [2]. In Thailand, the two common species reported in northern and southern parts are *L*. *(Mundinia) martiniquensis* and *L*. *(Mundinia) orientalis* which are the causative agents of VL, CL and DCL [3–11]. The disease is transmitted through the bites of infected sandflies [12]. Approximately 12 to 15 million people are infected and 350 million are at risk worldwide [13]. The geographical distribution of leishmaniasis is wide-ranging with concentrations in South Asia, North Africa, Middle East, Latin America and the Caribbean [14].

Clinical manifestations of leishmaniasis are diverse, comprising varying forms of diseases: cutaneous leishmaniasis (CL), disseminated cutaneous leishmaniasis (DCL), mucocutaneous leishmaniasis (MCL), and visceral leishmaniasis (VL) [15]. CL is a localized skin nodule at the bite site of a sandfly which progresses to papule and ulcer, but usually self-healing [15,16]. DCL manifests as disseminated skin lesions at various body parts other than the bite site (16). MCL is a disease that ulcerates mucosal tissue, generally invading nasal mucosa, but can involve the lips, cheeks, soft palate, pharynx and larynx in some cases [16,17]. VL, also known as ‘kala-azar’, is a severe fatal disease usually represented by fever, fatigue, weight loss, lymphadenopathy, hepatosplenomegaly and anemia [15,17].

Our previous studies reported both symptomatic and asymptomatic VL among individuals both positive and negative for HIV [3]. This study described one index case of CL in an immunocompetent male patient. A cross-sectional study of prevalence was conducted and associated factors of *Leishmania* infection were identified among those who were related to patients residing in urban and rural areas of Chiang Rai Province. The potential sandfly vector and animal reservoirs in the affected areas were also investigated.

## Methods

### Ethics statement

The research protocol was approved by the Ethics Committee of the Royal Thai Army Medical Department; approval number S005h/59. The written informed consent was obtained from all participants and the written assent form was obtained from the parent/guardian of children age younger than 18 years.

### History of the index case

In April 2015, an 18-year-old Thai male living in a semi-boarding vocational school, Mueang Chiang Rai District, Chiang Rai Province sustained a motorcycle accident resulting in a forehead laceration, sized 7 to 9 cm in length. The wound was cleansed, sutured with nine stitches, and dressed properly. Seven days later, on a follow-up visit, his wound had not closed and discharge was oozing from the wound. No further investigation or treatment was provided concerning his unhealed wound. In May 2015, he visited the hospital with a complaint of a soft nodule at the same site of his forehead wound. The nodule was incised and drained, revealing pus inside. The incised wound was sutured with three stitches. Following that, his wound became chronic with constantly oozing pus. Two months later, his chronic wound was biopsied revealing amastigotes of *Leishmania* spp. Subsequently, nested ITS1-PCR and nucleotide sequencing showed the infection was caused by *L*. *martiniquensis*. Physical examination did not show other signs and symptoms of VL. In September 2015, miltefosine 50 mg was given twice daily for 28 days, and then he revisited the hospital for a check-up the following month. The wound on his forehead was completely healed.

### Study design and study population

The study began with a single index case of CL reported in August 2015, then an outbreak investigation was subsequently conducted from September to November 2015 to identify the prevalence and associated risk factors of *Leishmania* infection among immunocompetent individuals who had previously contacted or resided within the housing area of the index case. The study areas included 1) students at a semi-boarding vocational school, an urban area, Mueang Chiang Rai District where the index case had been studied and 2) hill tribe villagers residing in the hometown of the index case, a remote and mountainous area, Wiang Kaen District, Chiang Rai Province.

Chiang Rai is the northernmost province of Thailand, located about 780 km from Bangkok, bordered by Myanmar to the north and Lao PDR to the east. Various areas of the province, especially the western rim, characterized by mountains and highland plateaus with an average height of 1,500 to 2,000 m above sea level. Eastern areas of the province comprise mostly lowland plains. Forest areas cover about 32.42% of the total area, and the average temperature is 24°C. Locations of the two study areas are as described below. First, Mueang Chiang Rai District, Chiang Rai Province is located at the center of the province, and constitutes the administrative hub of Chiang Rai, one of the largest cities in Thailand’s northern region. The semi-boarding school is located around 20 km from the center of Mueang Chiang Rai District. The dormitory is situated in the lush forest-garden area of the school with evident cracks in the wall building. Second, Wiang Kaen District is located on the northeastern edge of Chiang Rai Province, mostly outlined by high mountains and plateaus with a river valley in between. Villagers residing in this area are hill tribes, comprising Hmong, Lahu, Akha, Lisu and others. The site of the study village is located in a remote and mountainous area of the district where housing styles of hill tribes, predominantly Hmong, are mostly brick, wooden or mud-walled houses with soil flooring. Most houses are located in plantations mixed with forested areas.

### Definition

Symptomatic VL is defined as individuals presenting a history of fever lasting at least two weeks with splenomegaly. A single or combined clinical characteristic(s) of the following may be observed: hepatomegaly, weight loss, anemia, leucopenia, thrombocytopenia and hypergammaglobulinemia. Detection of the parasites must be confirmed under microscopic examination or by PCR assay using any clinical samples, i.e., bone marrow aspirates, lymph nodes, buffy coat or other biopsy samples.

Symptomatic CL is defined as individuals presenting skin lesions, mainly ulcers, on exposed parts of the body. Detection of the parasites must be confirmed under microscopic examination of skin biopsies or by PCR assay using those biopsies.

Asymptomatic *Leishmania* infection is defined as individuals experiencing no symptoms of CL/VL but presenting a positive test by DAT or PCR assays.

Seropositivity of *Leishmania* infection is defined as the detection of antibodies among individuals experiencing the infection and being either symptomatic or asymptomatic.

### Collecting and preparing human and animal blood samples

Eight mL of human whole blood samples were collected in EDTA anticoagulant tubes. Samples were centrifuged at 900 × g for 10 minutes to separate plasma and buffy coat and then kept at –20°C until used. Additionally, blood samples of animals at both the semi-boarding school and hill tribes’ households were also collected using the same protocol as that of human blood. Those animals included water buffaloes (*Bubalus bubalis*), dogs (*Canis familiaris*), cats (*Felis catus*), black rats (*Rattus rattus*), house lizards (*Hemidactylus platyurus*), toads (*Bufo asper*) and poultry (as shown in Table 1).

**Table 1:** Baseline characteristics of studied population.

Characteristics	Vocational school (Mueang Chiang Rai District) n (%)	Wiang Kaen District n (%)	Total n (%)
**Site**			
Wiang Kaen District	-	130 (33.2)	130 (33.2)
Vocational school (Mueang Chiang Rai District)	262 (66.8)	-	262 (66.8)
**Gender**			
Male	170 (64.9)	67 (51.5)	237 (60.5)
Female	92 (35.1)	63 (48.5)	155 (39.5)
**Nationalities**			
Thai	236 (90.1)	57 (43.8)	293 (74.7)
Laotian	3 (1.1)	69 (53.1)	72 (18.4)
Myanmar	4 (1.5)	1 (0.8)	5 (1.3)
Chinese	2 (0.8)	0 (0)	2 (0.5)
Other	17 (6.5)	3 (2.3)	20 (5.1)
**Hill tribes**			
Hmong	19 (7.2)	1 (0.8)	20 (5.1)
Lahu	75 (28.6)	4 (3.1)	79 (20.2)
Akha	51 (19.5)	0 (0)	51 (13.0)
Lisu	82 (31.3)	124 (95.4)	206 (52.5)
Others	35 (13.4)	1 (0.8)	36 (9.2)
**Occupation**			
Student	258 (98.5)	55 (42.3)	313 (79.8)
Farmer/rubber planter	1 (0.4)	60 (46.2)	61 (15.6)
Unemployed	0 (0)	7 (5.4)	7 (1.8)
Other	3 (1.1)	8 (6.1)	11 (2.8)
**History of working aboard**			
Yes	36 (13.7)	2 (1.5)	38 (9.7)
No	221 (84.4)	122 (93.9)	343 (87.5)
Unknown	5 (1.9)	6 (4.6)	11 (2.8)
**History of working in other provinces**			
Yes	190 (72.5)	15 (11.5)	205 (52.6)
No	70 (26.7)	109 (83.9)	179 (45.4)
Unknown	2 (0.8)	6 (4.6)	8 (2.0)
**History of sandfly bite**			
Yes	1 (0.4)	3 (2.3)	4 (1.0)
No	74 (28.2)	103 (79.2)	177 (45.2)
Unknown	187 (71.4)	24 (18.5)	211 (53.8)
**History of blood recipient**			
Yes	4 (1.5)	2 (1.5)	6 (1.5)
No	247 (94.3)	125 (96.2)	372 (94.9)
Unknown	11 (4.2)	3 (2.3)	14 (3.6)
**History of drug use**			
Yes	5 (1.9)	2 (1.5)	7 (1.8)
No	246 (93.9)	122 (93.9)	368 (93.8)
Unknown	11 (4.2)	6 (4.6)	17 (4.4)
**Animal in community**			
Yes	156 (59.5)	57 (43.8)	213 (54.5)
No	96 (36.6)	66 (50.8)	162 (41.3)
Unknown	10 (3.8)	7 (5.4)	17 (4.2)
**Animal raising**			
**Pigs**			
Yes	29 (11.1)	15 (11.5)	44 (11.2)
No	233 (88.9)	115 (88.5)	348 (88.8)
**Dogs**			
Yes	94 (35.9)	23 (17.7)	117 (29.8)
No	168 (64.1)	107 (82.3)	275 (70.2)
**Cats**			
Yes	32 (12.2)	20 (15.4)	52 (13.3)
No	230 (87.8)	110 (84.6)	340 (86.7)
**Chickens**			
Yes	38 (14.5)	26 (20.0)	64 (16.3)
No	224 (85.5)	104 (80.0)	328 (83.7)
**Ducks**			
Yes	5 (1.9)	4 (3.1)	9 (2.3)
No	257 (98.1)	126 (96.9)	383 (97.7)
**Housing environments**			
**Dark housing**			
Yes	60 (22.9)	67 (51.5)	127 (32.4)
No	202 (77.1)	63 (48.5)	265 (67.6)
**Untidy housing**			
Yes	101 (38.5)	43 (33.1)	144 (36.7)
No	161 (61.5)	87 (66.9)	248 (63.3)
**Soil flooring**			
Yes	88 (33.6)	37 (28.5)	125 (31.9)
No	174 (66.4)	93 (71.5)	267 (68.1)
**Having termite mounds**			
Yes	63 (24.1)	22 (16.9)	85 (21.7)
No	199 (75.9)	108 (83.1)	307 (78.3)
**Having animal enclosures**			
Yes	104 (39.7)	58 (44.6)	162 (41.3)
No	158 (60.3)	72 (55.4)	230 (58.7)
**Having trees**			
Yes	82 (31.3)	33 (25.4)	115 (29.4)
No	180 (68.7)	97 (74.6)	277 (70.6)
**Having unused buildings**			
Yes	49 (18.7)	5 (3.8)	54 (13.8)
No	213 (81.3)	125 (96.2)	338 (86.2)
**Having timbers and logs**			
Yes	66 (25.2)	46 (35.4)	112 (28.6)
No	19 (74.8)	84 (64.6)	280 (71.4)
**Having mammals**			
Yes	101 (38.5)	87 (66.9)	188 (47.9)
No	161 (61.5)	43 (33.1)	204 (52.1)
**Protective measures**			
**Lotion repellent**			
Yes	191 (72.9)	48 (36.9)	239 (61.0)
No	71 (27.1)	82 (63.1)	153 (39.0)
**Spray repellent**			
Yes	163 (62.2)	31 (23.8)	194 (49.5)
No	99 (37.8)	99 (76.2)	198 (50.5)
**Fog repellent**			
Yes	202 (77.1)	56 (43.1)	258 (65.8)
No	60 (22.9)	74 (56.9)	134 (34.2)
**Long sleeve shirt**			
Yes	158 (60.3)	82 (63.1)	240 (61.2)
No	104 (39.7)	48 (36.9)	152 (38.8)
**Cloth spray**			
Yes	44 (16.8)	18 (13.8)	62 (15.8)
No	218 (83.2)	112 (86.2)	330 (84.2)
**Bed net**			
Yes	100 (38.2)	89 (68.5)	189 (48.2)
No	162 (61.8)	41 (31.5)	203 (51.8)
**Spray Bed net**			
Yes	49 (18.7)	20 (15.4)	69 (17.6)
No	213 (81.3)	110 (84.6)	323 (82.4)
**Avoid night work**			
Yes	197 (75.2)	63 (48.5)	260 (66.3)
No	65 (24.8)	67 (51.5)	132 (33.7)
**Avoid anthill**			
Yes	132 (50.4)	49 (37.7)	181 (46.2)
No	130 (49.6)	81 (62.3)	211 (53.8)
**Tidy house**			
Yes	204 (77.9)	62 (48.5)	267 (68.1)
No	58 (22.1)	67 (51.5)	125 (31.9)
**Tidy environment**			
Yes	205 (78.2)	63 (48.5)	268 (68.4)
No	57 (21.8)	67 (51.5)	124 (31.6)
**Avoid carrier**			
Yes	176 (67.2)	51 (39.3)	227 (57.9)
No	86 (32.8)	79 (60.7)	165 (42.1)

### Screening for *Leishmania* antibodies using the DAT

The plasma of immunocompetent participants and animals were tested for *Leishmania* antibodies by the DAT using DAT kit (KIT Biomedical Research, Amsterdam, the Netherlands) following manufacturer instruction. The positive control was retrieved from the plasma of confirmed VL cases using PCR, while the negative control was retrieved from the plasma of healthy individuals. Positive titers were detected at value ≥1:100 [18].

### Detecting *Leishmania* DNA in buffy coat

Two hundred μL buffy coat of both humans and animals were extracted using Gen UPTM gDNA Kit (BiotechRabbit, Hennigsdorf, Germany). The final volume of 40 μL was eluted and preserved at -20°C. The ITS1 region of the small subunit ribosomal RNA (ss-rRNA) of *Leishmania* was amplified using a nested PCR method. During the primary PCR process, LITSR and L5.8 were used as primers to amplify 319–348 amplicons [19]. During the secondary PCR process, LITSR2 and L5.8S inner were used as primers [18]. The 25 μL of PCR master mix for buffy coat comprised 12.5 pmol of each primer, 0.2 mM of dNTP (Promega, USA), 1.5 mM of MgCl_2_, 1x PCR buffer and 1 U of Taq DNA polymerase (Promega, USA). Four μL and 1 μL of DNA templates were used during primary and secondary reactions, respectively. The positive control was the DNA of *L*. *martiniquensis* promastigotes. The whole process consisted of predenaturation at 94°C for 3 minutes, 35 cycles of 94°C of denaturation for 1 minute, 54°C of annealing for 30 seconds, 72°C of extension for 30 seconds and 72°C of final extension for 5 minutes. PCR products were separated by electrophoresis and visualized by Molecular Imager Gel Doc XR+ System with Imager Lab 3.0 Program (BioRad, USA).

### Sandflies collection, identification, and *Leishmania* DNA detection

In November 2015, trapped sandflies in the two study areas were collected indoors (houses with cracked walls) and outdoors using CDC light traps from 18.00 to 06.00 hr for three days. Outdoor areas included animal enclosures, termite mounds, stacks of bricks and wood, bamboo trees, banana trees, palm trees etc. Species of sandflies were identified based on morphological characteristics [20,21]. Female sandflies were kept in 1.5 microcentrifuge tubes containing 70% alcohol and were labeled accordingly for further molecular studies of leishmania infection.

To identify potential vectors, females were evaluated for *Leishmania* DNA using nested ITS1-PCR. The individual sandfly body was homogenized with a sealed Pasteur pipette in 1.5-ml tubes. One hundred mL of phosphate-buffered saline was added and samples were placed at 65°C for 30 minutes. Following the addition of 100 μL of 3M potassium acetate (pH 7.2), the homogenates were incubated on ice for 30 minutes and centrifuged for 15 minutes at 13,000 x g, and supernatants were collected. DNA was precipitated by adding 600 μL of 100% ethanol. DNA pellets were resuspended in 50 μL of 0.5x Tris-EDTA (TE) (pH 8.0) then QIAamp genomic DNA Kits (Venlo, the Netherlands) were used to detect DNA by nested ITS1-PCR.

### Questionnaire

The standardized questionnaire included demographic data, clinical symptoms and risk behaviors. The data were collected by face-to-face interview.

### Statistical analysis

The program used for statistical analysis was STATA, Version SE14 (Stata Corporation, College Station, TX, USA). Possible associated factors of *Leishmania* infections were analyzed using univariate analysis. Factors that were statistically significant by univariate analysis (*p* <0.05 or *p* <0.25) were included for multivariate analysis using the ‘Enter’ function to eliminate confounding factors. Odds ratio (OR) and 95% confidential interval (CI) were calculated for both univariate and multivariate analyses. Factors indicating *p <*0.05 by multivariate analysis were considered risk factors of *Leishmania* infection.

## Results

### Baseline characteristics of the population

In 2015, at the time of the study, a total of 770 participants were actively residing in the study areas, 500 students in the vocational school and 270 hill tribe people residing in the village. A total of 737 (95.7%) participants, 447 (89.4%) from the vocational school, and 270 (100%) from the rural village were screened for eligibility to join the study using a standardized questionnaire and physical examination. Of those 434 (58.9%) participants, 164 (36.7%) from the rural village and 270 (100%) from the vocational school, reported a history of close contact to the index case and none had any suspected symptoms of leishmaniasis. Among those eligible for study enrollment, a total of 392 (90.3%) immunocompetent individuals, 130 (79.3%) from the rural village, and 262 (97.0%) from the vocational school were enrolled in this study. Of 262, 6 students originally came from Wiang Kaen District.

The characteristics of the 392 participants from the two study areas, a mountainous and rural village in Wiang Kaen District, and a semi-boarding vocational school, an urban area in Mueang Chiang Rai District are shown in Table 1. Of these, 237 (60.5%) were male and 177 (45.2%) were 0 to 17 years of age. Hmong people constituted 206 (52.6%) members of the population. Most were students (79.9%) and originally came from Wiang Kaen District (66.8%). Most had no existing comorbidities and showed no clinical manifestations of VL/CL. Of these, 9.7% had a history of working abroad while 52.7% had a history of working in other provinces. History of having been bitten by sandflies was approximately 1%; while history of blood transfusion was 1.5% and recreational drug users comprised 1.8%.

### Prevalence of *Leishmania* infection among humans

Of 392 participants recruited from the two study areas, 28 (7.1%) were positive for *Leishmania* infection. Ten of the cases were from Amphoe Mueang Chiang Rai District and 18 were from Wiang Kaen District. Of 28, 15 (53.6%) were DAT positive while 21 (75.0%) were positive for nested ITS1-PCR. Species identification revealed that 1 (4.8%) was *L*. *martiniquensis*, 12 (57.1%) were *L*. *orientalis* and 8 (38.1%) were *Leishmania* spp.

### Animal reservoir

To identify potential animal reservoirs, plasma, collected from two water buffaloes (*Bubalus bubalis*), six dogs (*Canis familiaris*), six cats (*Felis catus*), one rat (*Rattus rattus*), two house lizards (*Hemidactylus platyurus*), one toad (*Bufo asper*) and 29 poultry samples, was tested for antibodies against *Leishmania* infection using DAT and *Leishmania* DNA by nested ITS1-PCR. Of these, only one rat (*Rattus rattus*) captured at the vocational school was PCR positive for DNA of *L*. *martiniquensis*. Additionally, plasma collected from two water buffaloes, two dogs, and one black rat at the vocational school revealed antibodies at variable titers, 1:100 to 1:>3200. In addition, at Wiang Kaen District, plasma of three dogs, four cats, and nine poultry samples revealed antibodies at variable titers, 1:100 to 1:>3200 (Table 2).

**Table 2:** Detection of *Leishmania* DNA among animals and trapped sandflies in the study areas of a vocational institute, an urban area and a mountainous rural area in Wiang Jaen District, Chiangrai Province, northern Thailand. Direct Agglutination Test (DAT) was used to detect antibodies in animal plasma.

Sample	Total	Vocational school			Wiang Kaen District		
		No sample	PCR positive	DAT positive	No sample	PCR positive	DAT Positive
Sandflies	76						
*Sergentomyia gemmea*		64	7*	N/A	0	0	N/A
*Phlebotomus stantoni*		8	1**	N/A	4	0	N/A
Buffaloes (*Bulalus bubalis*)	2	2	0	2^#^	0	0	0
Dogs (*Canis faniliaris*)	6	2	0	2^##^	4	0	3 (>1:100)
Cats (*Felis catus*)	6	0	0		6	0	4 (>1:100)
Black rat *(Rattus rattus*)	1	1	1***	1^###^	0	0	0
House lizard (*Hemidactylus platyurus*)	2	1	0	N/A	1	0	N/A
Toad (*Bufo asper*)	1	0	0	N/A	1	0	N/A
Poultry	29	0	0	0	29	0	9^$^

*L. *orientalis* = 6 samples, *L*. *donovani/L*. *infantum* = 1 sample,

**L. *orientalis* = 1 sample

***L. *martiniquensis* = 1 sample

N/A = not applicable

DAT

^#^ >1:3200 = 2 cases,

^##^>1:1600 = 1 case, >1:3200 = 1 case, and

^###^>1:100 = 1 case

^$^1:100 = 1 case, 1:200 = 3 cases, 1:400 = 1 case and ≥1:800 = 4 cases

### Sandfly identification and Leishmania DNA detection

A total of 76 sandflies were collected at Wiang Kaen District and the semi-boarding vocational school in Mueang Chiang Rai District, Chiang Rai Province. Morphological identification revealed 64 *Sergentomyia* (*Neophlebotomus*) *gemmea* and 8 *Phlebotomus* (*Anaphlebotomus*) *stantoni* captured at the semi-boarding vocational school. Moreover, 4 *P*. *stantoni* samples were identified in Wiang Kaen District. As shown in Table 2, at the semi-boarding vocational school, 7 (10.9%) *S*. *gemmea* was PCR positive for DNA of *L*. *orientalis* while 1 (12.5%) *P*. *stantoni* was PCR positive for DNA of *L*. *martiniquensis*. However, at Wiang Kaen District, four captured *P*. *stantoni* were PCR negative for *Leishmania* DNA.

### Associated risk factors of *Leishmania* infection

Univariate and multivariate analysis results for risk factors of *Leishmania* infection are shown in Table 3. *Leishmania* infection was associated with being female (AOR 2.52, 95%CI 1.01–6.26), increasing age (AOR 1.05, 95%CI 1.02–1.08), having animal enclosures (AOR 3.04, 95%CI 1.13–8.22), being exposed to termite mounds (AOR 3.74, 95%CI 1.11–12.58) and having domestic animals in a housing area (AOR 7.11, 95%CI 2.08–24.37) after having adjusted for confounding factors with dark housing, soil flooring, having termite mound nearby residential house, using repellent, tidy house and tidy environment.

**Table 3:** Univariate and multivariate analysis of risk factors of *Leishmania* infection among patients in Chiang Rai Province, Thailand (n = 392).

Characteristics	Leishmania infection		Univariate analysis			Multivaraite analysis		
	Negative n (%)	Positive n (%)	Crude OR	95% CI	*P*-value	Adjusted OR	95% CI	*P*-value
Gender								
Male	225 (94.94)	12 (5.06)	1.00			1.00		
Female	139 (89.68)	16 (10.32)	2.16	0.99–4.70	0.053	2.52	1.01–6.26	0.047
Age (mean (sd))	19.40 (10.96)	30.78 (19.59)	1.05	1.02–1.07	< 0.001	1.05	1.02–1.08	0.003
Nationality								
Thai	276 (94.20)	17 (5.80)	2.03	0.92–4.50	0.081			
Other	88 (88.89)	11 (11.11)	1.00					
Occupation								
Students	297 (94.89)	16 (5.11)	3.32	1.50–7.36	0.003			
Others	67 (84.81)	12 (15.19)	1.00					
Site								
Wiang Kaen District	112 (86.15)	18 (13.85)	4.05	1.81–9.05	0.001			
Vocational school (Amphoe Mueang Chiang Rai District)	252 (96.18)	10 (3.82)	1.00					
Hill tribe								
Lisu	180 (87.38)	26 (12.62)	13.29	3.11–56.81	<0.001			
Other	184 (98.92)	2 (1.08)	1.00					
Pig raising								
Yes	38 (86.36)	6 (13.64)	2.34	0.89–6.13	0.084			
No	326 (93.68)	22 (6.32)	1.00					
Dog raising								
Yes	111 (94.87)	6 (5.13)	0.62	0.24–1.57	0.316			
No	253 (92.00)	22 (8.00)	1.00					
Cat raising								
Yes	51 (98.08)	1 (1.92)	0.23	0.03–1.71	0.150			
No	313 (92.06)	27 (7.94)	1.00					
Chicken raising								
Yes	54 (84.38)	10 (15.63)	3.19	1.40–7.28	0.006			
No	310 (94.51)	18 (5.49)	1.00					
Duck raising								
Yes	5 (55.56)	4 (44.44)	11.97	3.02–47.48	<0.001			
No	359 (93.73)	24 (6.27)	1.00					
Mammal pets								
Yes	170 (90.43)	18 (9.57)	2.05	0.92–4.57	0.078			
No	194 (95.10)	10 (4.90)	1.00					
Dark housing								
Yes	113 (88.98)	14 (11.02)	2.22	1.02–4.81	0.043	1.95	0.77–4.93	0.159
No	251 (94.72)	14 (5.28)	1.00			1.00		
Untidy house								
Yes	134 (93.06)	10 (6.94)	0.95	0.43–2.13	0.907			
No	230 (92.74)	18 (7.26)	1.00					
Soil flooring								
Yes	110 (88.00)	15 (12.00)	2.66	1.23–5.79	0.013	1.56	0.56–4.26	0.388
No	254 (95.13)	13 (4.87)	1.00			1.00		
Having termite mound nearby residential house								
Yes	75 (88.24)	10 (11.76)	2.14	0.95–4.83	0.067	2.66	0.83–8.56	0.100
No	289 (94.14)	18 (5.86)	1.00			1.00		
Having animal enclosures (within 200 meters around the house)								
Yes	142 (87.65)	20 (12.35)	3.91	1.68–9.11	0.002	3.04	1.13–8.22	0.028
No	222 (96.52)	8 (3.48)	1.00			1.00		
Trees with large bark in housing area								
Yes	102 (88.70)	13 (11.30)	2.23	1.02–4.84	0.044			
No	262 (94.58)	15 (5.42)	1.00					
Unused small huts in housing area								
Yes	48 (88.89)	6 (11.11)	1.79	0.69–4.65	0.228			
No	316 (93.49)	22 (6.51)	1.00					
Timbers and logs in housing area								
Yes	100 (89.29)	12 (10.71)	1.98	0.90–4.33	0.087			
No	264 (94.29)	16 (5.71)	1.00					
Using repellent								
Yes	137 (89.54)	16 (10.46)	1.00			1.00		
No	227(94.98)	12 (5.02)	1.75	0.72–4.22	0.214	0.41	0.13–1.28	0.125
Wearing long sleeve shirt								
Yes	226 (94.17)	14 (5.83)	1.00					
No	138 (90.79)	14 (9.21)	1.63	0.76–3.54	0.210			
Fog repellent								
Yes	246 (95.35)	12 (4.65)	1.00					
No	118 (88.06)	16 (11.94)	2.78	1.27–6.06	0.010			
Insect repellent spray on cloth								
Yes	62 (100.00)	0 (0.00)						
No	302 (91.52)	28 (8.48)						
Bed net use								
Yes	178 (94.18)	11 (5.82)	1.00					
No	186 (91.63)	17 (8.37)	1.48	0.67–3.24	0.329			
Bed net spray								
Yes	69 (100.00)	0 (0.00)						
No	295 (91.33)	28 (8.67)						
Avoid working at night								
Yes	247 (95.00)	13 (5.00)	1.00					
No	117 (88.64)	15 (11.36)	2.43	1.12–5.28	0.024			
Tidy house								
Yes	255 (95.51)	12 (4.49)	1.00			1.00		
No	109 (87.20)	16 (12.80)	3.12	1.43–6.81	0.004	1.53	0.38–6.18	0.550
Tidy environment								
Yes	255 (95.15)	13 (4.85)	1.00			1.00		
No	109 (87.90)	15 (12.10)	2.70	1.24–5.86	0.012	0.85	0.20–3.59	0.829
Avoid termite mounds								
Yes	177 (97.79)	4 (2.21)	1.00			1.00		
No	187 (88.63)	24 (11.37)	5.68	1.93–16.69	0.107	3.74	1.11–12.58	0.033
Avoid exposure to carrier animals								
Yes	222 (97.80)	5 (2.20)	1.00			1.00		
No	142 (86.06)	23 (13.94)	7.19	2.67–19.35	<0.001	7.11	2.08–24.37	0.002

After separately analyzing the sites, it was found that associated factors of *Leishmania* infection in Wiang Kaen District were increasing age (AOR 1.05, 95%CI 1.01–1.10), having termite mound near residential areas (AOR 44.10, 95%CI 2.88–676.37), having animal enclosure near housing areas (AOR 6.94, 95%CI 1.17–41.01), not avoid termite mounds (AOR 16.51, 95%CI 1.31–207.64) and not avoid exposure to carrier animals (AOR11.76, 95%CI 1.00–138.21) after adjusted for confounding factors. For Amphoe Mueang Chiang Rai District, associated factors were dark housing (AOR 5.01, 95%CI 1.16–21.64) and not avoid exposure to carrier animals (AOR 8.33, 95%CI 1.36–50.90) after adjusted for confounding factors. Tables 4 and 5 staged the univariate and multivariate analysis of risk factors of *Leishmania* infection in Wiang Kaen and Amphoe Mueang Chiang Rai Districts, respectively.

**Table 4:** Univariate and multivariate analysis of risk factors of *Leishmania* infection among patients in Amphoe Wiang Kaen District, Chiang Rai, Thailand (n = 130).

Characteristics	Outcomes		Univariate analysis			Multivaraite analysis		
	Uninfected n (%)	Infected n (%)	Crude OR	95% CI	*P*-value	Adjusted OR	95% CI	*P*-value
Gender								
Male	61 (91.04)	6 (8.96)	1.00			1.00		
Female	51 (80.95)	12 (19.05)	2.39	0.84–6.82	0.103	2.56	0.58–11.31	0.215
Age (mean (sd))	19.40 (10.96)	30.78 (19.59)	1.05	1.02–1.08	<0.001	1.05	1.01–1.10	0.012
Occupation								
Students	49 (89.09)	6 (10.91)	1.56	0.55–4.44	0.409			
Others	63 (84.00)	12 (16.00)	-	-	-			
Nationality								
Thai	49 (85.96)	8 (14.04)	0.97	0.36–2.65	0.956			
Other	63 (86.30)	10 (13.70)	1.00					
Hill tribe								
Lisu	107 (86.29)	17 (13.71)	0.79	0.09–7.22	0.838			
Other	5 (83.33)	1 (16.67)	1.00					
History of working aboard (n = 257)								
Yes	35 (97.22)	1 (2.78)	1.13	0.32–3.93	0.850			
No	212 (95.93)	9 (4.07)	1.00					
History of working in other provinces (n = 260)								
Yes	183 (96.32)	7 (3.68)	0.66	0.31–1.44	0.302			
No	67 (95.71)	3 (4.29)	1.00					
History of sandfly bite (n = 75)								
Yes	1 (100.00)	0 (0.00)	4.46	0.45–44.31	0.202			
No	66 (89.19)	8 (10.81)	1.00					
Pig raising								
Yes	12 (80.00)	3 (20.00)	1.67	0.42–6.60	0.467			
No	100 (86.96)	15 (13.04)	1.00					
Dog raising								
Yes	21 (91.30)	2 (8.70)	0.54	0.12–2.54	0.437			
No	91 (85.05)	16 (14.95)	1.00					
Cat raising								
Yes	19 (95.00)	1 (5.00)	0.29	0.04–2.30	0.240			
No	93 (84.55)	17 (15.45)	1.00					
Chicken raising								
Yes	20 (76.92)	6 (23.08)	2.30	0.77–6.86	0.135			
No	92 (88.46)	12 (11.54)	1.00					
Duck raising								
Yes	0 (-)	0 (-)	6.88	0.90–52.59	0.063			
No	112 (86.15)	18 (13.85)	1.00					
Dark housing								
Yes	59 (88.06)	8 (11.94)	0.72	0.26–1.96	0. 518	0.42	0.08–2.21	0.305
No	53 (84.13)	10 (15.87)	1.00			1.00		
Untidy house								
Yes	38 (88.37)	5 (11.63)	0.75	0.25–2.26	0.608			
No	74 (85.06)	13 (14.94)	1.00					
Mammal pet								
Yes	74 (85.06)	13 (14.94)	1.34	0.44–4.02	0.608			
No	38 (88.37)	5 (11.63)	1.00					
Soil flooring								
Yes	28 (75.68)	9 (24.32)	3.00	1.08–8.30	0.034	0.31	0.05–1.93	0.208
No	84 (90.32)	9 (9.68)	1.00			1.00		
Having termite mound nearby residential house								
Yes	15 (68.18)	7 (31.82)	4.12	1.38–12.27	0.011	44.10	2.88–676.37	0.007
No	97 (89.81)	11 (10.19)	1.00			1.00		
Having animal enclosures (within 200 meters around the house)								
Yes	43 (74.14)	15 (25.86)	8.02	2.19–29.34	0.002	6.94	1.17–41.01	0.033
No	69 (95.83)	3 (4.17)	1.00			1.00		
Trees with large bark in housing area								
Yes	25 (75.76)	8 (24.24)	2.78	0.99–7.80	0.052			
No	87 (89.69)	10 (10.31)	1.00					
Unused small huts in housing area								
Yes	2 (40.00)	3 (60.00)	11.0	1.70–71.28	0.012			
No	110 (88.00)	15 (12.00	1.00					
Timbers and logs in housing area								
Yes	37 (80.43)	9 (19.57)	1.29	0.32–5.12	0.722			
No	75 (89.29)	9 (10.71)	1.00					
Using repellent								
Yes	44 (91.67	4 (8.33)	1.00			1.00		
No	68 (82.93))	14 (17.07)	2.26	0.70–7.33	0.172	0.69	0.03–14.41	0.808
Wearing long sleeve shirt								
Yes	76 (92.68)	6 (7.32))	1.00					
No	36 (75.00)	12 (25.00	4.22	1.47–12.15	0.008			
Fog repellent								
Yes	51 (91.07)	5 (8.93))	1.00			1.00		
No	61 (82.43)	13 (17.57	2.17	0.73–6.51	0.165	0.41	0.13–1.28	0.125
Insect repellent spray on cloth								
Yes	94 (83.93)	18 (16.07)	1.00					
No	18 (100.00)	0 (0.00)	-	-	-			
Bed net use								
Yes	80 (89.89)	9 (10.11)	1.00					
No	32 (78.05)	9 (21.95)	2.50	0.91–6.87	0.076			
Bed net spray								
Yes	20 (100.00)	0 (0.00)	1.00					
No	92 (83.64)	18 (16.36)	-	-	-			
Avoid working at night								
Yes	58 (92.06)	5 (7.94)	1.00					
No	54 (80.60)	13 (19.40)	2.79	0.93–8.36	0.066			
Tidy house								
Yes	57 (90.48)	6 (9.52)	1.00			1.00		
No	55 (82.09)	12 (17.91)	2.07	0.73–5.91	0.173	126003.60	0 -.	0.995
Tidy environment								
Yes	57 (90.48)	6 (9.52)	1.00			1.00		
No	55 (82.09)	12 (17.91)	2.07	0.73–5.91	0.173	7.89e-06	0 -.	0.995
Avoid termite mounds								
Yes	48 (97.96)	1 (2.04)	1.00			1.00		
No	64 (79.01)	17 (20.99)	12.75	1.64–99.16	0.015	16.51	1.31–207.64	0.030
Avoid exposure to carrier animals								
Yes	49 (96.08)	2 (3.92)	1.00			1.00		
No	63 (79.75)	16 (20.25)	6.22	1.37–28.36	0.018	11.76	1.00–138.21	0.050

**Table 5:** Univariate and multivariate analysis of risk factors of *Leishmania* infection among patients in Vocational school (Amphoe Mueang Chiang Rai District), Chiang Rai, Thailand (n = 262).

Characteristics	Outcomes		Univariate analysis			Multivaraite analysis		
	Uninfected n (%)	Infected n (%)	Crude OR	95% CI	*P*-value	Adjusted OR	95% CI	*P*-value
Gender								
Male	164 (96.47)	6 (3.53)	1.00			1.00		
Female	88 (95.65)	4 (4.35)	1.24	0.34–4.52	0.742	1.74	0.38–7.98	0.474
Age (mean (sd))	19.40 (10.96)	30.78 (19.59)	0.68	0.44–1.07	0.097	0.65	0.41–1.04	0.073
Occupation								
Students	248 (96.12)	10 (3.88)	0.13	0.02–0.75	0.022			
Others	4 (100.00)	0 (0.00)	-	-	-			
Nationality								
Thai	227 (96.19)	9 (3.81)	1.01	0.12–8.30	0.993			
Other	25 (96.15)	1 (3.85)	1.00					
Hill tribe								
Lisu	73 (89.02)	9 (10.98)	22.07	2.75–177.33	0.004			
Other	179 (99.44)	1 (0.56)	1.00					
History of working aboard (n = 124)								
Yes	0 (0.00)	2 (100.00)	1.13	0.32–3.93	0.850			
No	107 (87.70)	15 (12.30)	1.00					
History of working in other provinces (n = 124)								
Yes	10 (66.67)	5 (33.33)	0.66	0.31–1.44	0.302			
No	97 (88.99)	12 (11.01)	1.00					
History of sandfly bite (n = 106)								
Yes	2 (66.67)	1 (33.33)	4.46	0.45–44.31	0.202			
No	87 (84.47)	16 (15.53)	1.00					
Animal raising								
Yes	150 (96.15)	6 (3.85)	0.97	0.45–2.09	0.933			
No	94 (97.92)	2 (2.08)	1.00					
Pig raising								
Yes	26 (89.66)	3 (10.34)	3.73	0.91–15.29	0.068			
No	226 (97.00)	7 (3.00)	1.00					
Dog raising								
Yes	90 (95.74)	4 (4.26)	1.20	0.33–4.36	0.782			
No	162 (96.43)	6 (3.57)	1.00					
Cat raising								
Yes	32 (100.00)	0 (0.00)	-	-	-			
No	220 (95.65)	10 (4.35)	1.00					
Chicken raising								
Yes	34 (89.47)	4 (10.53)	4.27	1.15–15.93	0.030			
No	218 (97.32)	6 (2.68)	1.00					
Duck raising								
Yes	3 (60.00)	2 (40.00)	20.75	3.03–141.92	0.002			
No	249 (96.89)	8 (3.11)	1.00					
Dark housing								
Yes	54 (90.00)	6 (10.00)	5.50	1.50–20.19	0.010	5.01	1.16–21.64	0.031
No	198 (98.02)	4 (1.98)	1.00			1.00		
Untidy house								
Yes	96 (95.05)	5 (4.95)	1.63	0.46–5.76	0.452			
No	156 (96.89)	5 (3.11)	1.00					
Mammal pet								
Yes	96 (95.05)	5 (4.95)	1.63	0.46–5.76	0.452			
No	156 (96.89)	5 (3.11)	1.00					
Soil flooring								
Yes	82 (93.18)	6 (6.82)	3.11	0.85–11.32	0.085	2.43	0.49–12.	0.278
No	170 (97.70)	4 (2.30)	1.00			1.00		
Having termite mound near by residential house								
Yes	60 (95.24)	3 (4.76)	1.37	0.34–5.47	0.654	1.11	0.21–6.04	0.901
No	192 (96.48)	7 (3.52)	1.00			1.00		
Having animal enclosures (within 200 meters around the house)								
Yes	99 (95.19)	5 (4.81)	1.55	0.44–5.48	0.500	1.60	0.36–6.99	0.534
No	222 (96.52)	8 (3.48)	1.00			1.00		
Trees with large bark in housing area								
Yes	77 (93.90)	5 (6.10)	1.27	0.64–8.08	0.205			
No	715 (97.22)	5 (2.78)	1.00					
Unused small huts in housing area								
Yes	46 (93.88)	3 (6.12)	1.92	0.48–7.70	0.358			
No	206 (96.71)	7 (3.29)	1.00					
Timbers and logs in housing area								
Yes	63 (95.45)	3 (4.55)	1.29	0.32–5.12	0.722			
No	189 (96.43)	7 (3.57)	1.00					
Using repellent								
Yes	183 (95.81)	8 (4.19)	1.00			1.00		
No	69 (97.18)	2 (2.82)	0.66	0.14–3.20	0.609	0.30	0.06–1.44	0.133
Wearing long sleeve shirt								
Yes	150 (94.94))	8 (5.06)	1.00					
No	102 (98.08	2 (1.92)	0.37	0.77–1.77	0.212			
Fog repellent								
Yes	195 (96.53)	7 (3.47)	1.00					
No	57 (95.00)	3 (5.00)	1.47	0.37–5.85	0.588			
Insect repellent spray on cloth								
Yes	44 (100.00)	0 (0.00)	1.00					
No	208 (95.41)	10 (4.59)	-	-	-			
Bed net use								
Yes	98 (98.00)	2 (2.00)	1.00					
No	154 (95.06)	8 (4.94)	2.55	0.53–12.24	0.243			
Bed net spray								
Yes	49 (100.00)	0 (0.00)	1.00					
No	203 (95.31)	10 (4.69)	-	-	-			
Avoid working at night								
Yes	189 (95.94)	8 (4.06)	1.00					
No	63 (96.92)	2 (3.08)	0.750	1.16–3.62	0.720			
Tidy house								
Yes	198 (97.06)	6 (2.94)	1.00			1.00		
No	54 (93.10)	4 (6.90)	2.44	0.67–8.97	0.178	1.57	0.24–10.42	0.641
Tidy environment								
Yes	198 (96.59)	7 (3.41)	1.00			1.00		
No	54 (94.74)	3 (5.26)	2.70	1.24–5.86	0.012	0.93	0.12–7.04	0.944
Avoid termite mounds								
Yes	129 (97.73)	3 (2.27)	1.00			1.00		
No	123 (94.62)	7 (5.38)	1.00	0.62–9.68	0.202	1.31	0.24–7.19	0.757
Avoid exposure to carrier animals								
Yes	173 (98.30)	3 (1.70)	1.00			1.00		
No	79 (91.86)	7 (8.14)	6.22	1.37–28.36	0.018	8.33	1.36–50.90	0.022

## Discussion

Our previous studies on prevalence and risk factors of leishmaniasis have focused on patients with HIV/AIDS, a high risk group, in southern Thailand [18,22,23]. This is the first community-based study to investigate the prevalence and associated risk factors of *Leishmania* infection among immunocompetent individuals, indicating the situation of *Leishmania* infection in endemic areas of Thailand. This study addressed the epidemiology of leishmaniasis related to the index case of CL caused by *L martiniquensis* from an individual studying in a semi-boarding conventional school located in an urban area, Chiang Rai Province, northern Thailand. This study also confirmed that the two main species of *Leishmania* infection, *L martiniquensis*, and *L*. *orientalis* could be detected among immunocompetent individuals residing in both rural and urban areas of the north as well as those previously reported in the south. Other *Leishmania* species were also identified in the affected areas. Unfortunately, the PCR assay used in this study amplified ITS1 which limited species identification of *Leishmania*. In this study, serological and molecular diagnosis among participants was not in concordance. DAT was used for antibody detection which could not identify past and present infection. The pooled sensitivity and specificity rates of DAT among VL caused by *L*. *donovani/ L*. *infantum* were 96% (95% CI, 92–98) and 95% (CI, 95% 86–99), respectively [24]. However, DAT was very useful to detect antibody against *L*. *martiniquensis* and *L*. *orientalis* infection [18]. Whereas, ITS1-PCR could detect DNA which could indicate present infection. Our previous studies also showed similar results [18,23].

For risk factor analysis, being female had about a twice higher risk of contracting *Leishmania* infection. Time of exposure to sandfly bites among females was more likely higher than males because females spent time at home where conditions for breeding and resting of sandflies in their houses were favorable. Hmong hill tribes usually live at high altitudes where the weather is usually cold. Culturally, their residences are built using bricks, wood or mud-walls, often have no windows and are directly built on the soil. One of many risk factors of *Leishmania* transmission is housing characteristics including mud walls with cracks and holes and damp and dark houses creating favorable conditions for sandfly breeding and resting during the day [25]. Other risk factors were increasing age [26], having animal enclosures [27,28], being exposed to termite mounds [27,29,30] and having domestic animals [27,28,31] in a housing area that supported the results of this study.

In this study, *Leishmania* antibodies at variable titers were detected in two water buffaloes, two dogs and one black rat at the vocational school. Further at Wiang Kaen District, three dogs, four cats, and nine poultry samples also demonstrated antibodies at variable titers. However, the DNA of *Leishmania* was not detected in the buffy coat of these animals. Participants who raised dogs, pigs, ducks and chickens were more likely to test positive than those who did not. Dogs were reported as a reservoir of *Leishmania* parasites [32,33], while other animals were suspected to be possible reservoirs [34] and increased risk of canine leishmaniasis [35]. Additionally, conditions of animal enclosures were mostly damp and low hygiene areas which were shown to present a risk of leishmaniasis [36,37]. As such, avoiding contact with reservoirs and risky environments is protective against *Leishmania* infection. People who were exposed to termite mounds in the housing area had a greater tendency to contract *Leishmania* than those who did not. Termite mounds were previously reported as associated with kalar-azar transmission [38]. Related reports indicated phlebotomine sandflies hide in the ventilating shafts of anthills and only emerge during the humid evening of the rainy season [39].

In Europe and the Mediterranean Basin, dogs serve as an important reservoir host of zoonotic transmission of *L*. *infantum* [40–42]. In Thailand, the transmission of *L*. *martiniquensis* is most likely to involve a zoonotic cycle. DNA of *L*. *martiniquensis* was detected in the liver, spleen and blood of black rats (*Rattus rattus*) captured around a patient’s house in southern Thailand [3,43]. This study confirmed that black rats could serve as a natural animal reservoir of *L*. *martiniquensis* when DNA of *L*. *martiniquensis* was detected in the buffy coat of one black rat, captured in the area of the vocational school. Moreover, evidence of zoonotic transmission of CL caused by *L*. *martiniquensis* was reported in horses in Germany [44], bovines in Switzerland [45] and one horse in Florida, USA [46].

Not avoiding exposure to carrier animals was significant from analyzing from both Wiang Kaen District site and Amphoe Mueang Chiang Rai District site, as well as from analyzing on combined data from both sites, suggesting importance of carrier animals in the role of disease transmission which related to other factors such as having animal enclosure nearby residential areas. Carrier animals in these two areas included dogs and cats which were already presented in previous studies [32,34–36]. In these settings, due to rural lifestyles of the people, it was unavoidable to be exposed to carrier animals in residences where domestic animals were raised in the nearby areas.

Sandflies of the family Phlebotomidae consist of three main genera, *Phlebotomus* and *Sergentomyia* in the Old World and *Lutzomyia* in the New World. At present, not only are morphological characteristics used to identify sandflies species but also a few molecular markers, i.e., cytochrome b of mitochondrial DNA; ITS2 and the D8 domain of ribosomal DNA have been used in phylogenetic studies and DNA barcoding [47]. The genus *Phlebotomus* includes 11 subgenera distributed across Europe, Africa and Asia. Depaquit et al., (2015) identified *Phlebotomus* (*Anaphlebotomus*) *stantoni* captured in Southeast Asian countries, i.e., Thailand (Chiang Mai Province), Malaysia, and Vietnam using both methods. In this study, sandflies species were identified using morphological characteristics [20,21] which small number of *P*. *(Anaphlebotomus) stantoni* was also found in Chiang Rai Province, both in urban and mountainous areas. In addition, only one *P*. (*Anaphlebotomus*) *stantoni* collected at the vocational school was found positive for *L*. *martiniquensis* DNA. In addition, six *Sergentomyia* (*Neophlebotomus*) *gemmea* at the vocational school were positive for *L*. *orientalis* DNA while one *S*. *gemmea* was positive for *L*. *donovani/L*. *infantum* DNA. Unfortunately, due to the conserved region of the ITS1 of these two related species, the ITS1-PCR assay used in this study could not differentiate between *L*. *donovani* and *L*. *infantum*. In this study, captured sandflies were less abundant in number and species compared with a related study conducted by Sriwongphun et al. (2017). A total of 17 species of two genera of captured sandflies, *Sergentomyia* and *Phlebotomus*, were identified at Wiang Khan District. The first three most abundant species of sandflies were *S*. *punjabensis*, followed by *S*. *gemmea* and *S*. *barraudi* which exhibits a higher abundance in November than other months of the year. Additionally, *P*. *argentipes*, an important vector of VL in Bangladesh, India and Nepal [48] was also previously identified in Wiang Khan District. However, according to morphological characteristics, Depaquit et. al. (2019) raised the issue of misidentification of *Sergentomyia* species in Thailand. The key diagnosis of *S*. *gemmea* was described in Lewis (1978) [21]. However, some characteristics of *S*. *gemmea* including pharyngeal and cibarial armatures may be similar to others related species. It is important to identify ascoidal spurs, a distinct characteristic, in *S*. *gemmea* [49].

In conclusion, our study confirmed *L*. *martiniquensis* and *L*. *orientalis* infection among immunocompetent hosts in Chiang Rai Province, northern Thailand. Further studies to explore the environment, climate patterns, and lifestyles of patients, as well as their communities for prevention and control strategies, are recommended. Most importantly, to investigate new infection, disease progression, as well as self-clearance, a follow-up study of affected individuals in the study areas, should be performed.
